# The perception of the scope of oral and maxillofacial surgery and differentiation from similar specialities among dental students, medical students, trainee interns and pre-vocational junior doctors

**DOI:** 10.1007/s10006-024-01261-y

**Published:** 2024-05-27

**Authors:** Robert Mane, William Fox Sharpe-Davidson, Harsha De Silva, Joanne Jung Eun Choi

**Affiliations:** 1 Te Whatu Ora, Health New Zealand, New Zealand; 2 Sir John Walsh Research Institute, Faculty of Dentistry, University of Otago, 310 Great King Street, Dunedin, New Zealand

**Keywords:** Oral and maxillofacial surgery, Awareness, Perception, Differentiation, Recognition, Dental and Medical students

## Abstract

**Purpose:**

To explore the ability of Dental Students (DS), Medical Students (MS), Trainee Interns (TI) and Pre-vocational Junior Doctors (JD) in identifying procedures performed by Oral and Maxillofacial Surgery (OMS), the scope of practice (SOP) of OMS and ability to differentiate OMS from similar specialities.

**Methods:**

The study included 282 complete responses to a survey consisting of: 9 demographic questions, 11 OMS awareness, professional ambition, teaching and exposure, confidence in identifying and referring questions and 70 procedural/scenario questions across four domains. Collected data was qualitatively and statistically analysed using SPSS V29.

**Results:**

OMS awareness was limited. 92.2% reported None to Small amount of OMS teaching and exposure during university, with 66.0% preferring to have had a Fair to Significant amount. DS experienced more than medical respondents (2.1 vs. 1.5, *p* < 0.001). Most respondents reported No to Low confidence in identifying the SOP and procedures performed by OMS (67.7%) and referring to OMS (62.4%) compared to similar specialities (32.4% and 33.2%, respectively). 52.9% of procedures performed by OMS were correctly identified as being performed by OMS. The ability to identify the OMS SOP (8.7%) and differentiate OMS from similar specialities (5.0%) was low, however better among DS than medical respondents (14.9% vs. 6.3%, *p* = 0.002 and 12.2% vs. 2.2%, *p* < 0.001).

**Conclusion:**

The study has highlighted a deficit in the understanding of OMS with potential implications in the public and private healthcare sector. Identification of procedures, OMS SOP and ability to differentiate OMS from similar specialities is limited however slightly better among DS.

**Supplementary Information:**

The online version contains supplementary material available at 10.1007/s10006-024-01261-y.

## Introduction

Oral and Maxillofacial Surgery (OMS) involves the diagnosis and treatment (operative and non-operative) of patients with diseases, injuries and defects of the mouth, jaws and associated structures. This includes oral and maxillofacial pathology, trauma, dentoalveolar surgery, orthognathic, and relevant reconstructive surgery, and facial pain [1].

The scope of practice (SOP) of OMS appears frequently misunderstood amongst healthcare practitioners [2], often overlapping with similar professions including Otorhinolaryngology (ENT), Plastic and Reconstructive Surgery (Plastics), Ophthalmology and Dental. Unsurprisingly, an OMS-intended referral addressed to “*Jaw Plastic Surgeons*” or “*Maxilo-facial Dentists*” with inappropriate indications e.g. “*dental check/tooth decay*” is not uncommon. These deleterious misunderstandings may exacerbate healthcare inefficiency and increase health burden with delayed patient care, missed healthcare opportunities, wasted healthcare resources, inappropriate referrals, interprofessional confusion and frustration, interprofessional isolation and missed workforce and educational opportunities [3]. The multifactorial confusion may be impacted by various educational, professional, employment, hospital and individual factors.

SOP is individually and professionally dynamic [4], often impacted by key stakeholders including government groups, registration and credentialing bodies, training organisations, local hospital systems, population needs and individual training. A multi-tiered OMS system exists having developed alongside the specialty’s maturation with specialist registration through the Medical Council of New Zealand (MCNZ) and/or Dental Council of New Zealand (DCNZ). Surgeons may be dental-trained or dual-trained in medicine and dentistry and can register with University, College, or Board-certified qualifications, relevant Medical Council registration, or experiential backgrounds.

The Fellowship of the Royal College of Dental Surgeons (Oral and Maxillofacial Surgery) is the primary registerable Australasian qualification, with a minimum 4-years duration and requiring applicants to be dual-trained with completion of a full year of Surgery-in-General. OMS training units are positioned at four of the five national Advanced Trauma Centres. Educational agreements exist between these hospitals and the University of Auckland and University of Otago, which are responsible for all NZ medical and dental training. Only the Bachelor of Dental Surgery (BDS) curriculum has formal OMS teaching.

Unlike other specialities, OMS demand is unknown as the Ministry of Health does not collect OMS health statistics. Potentially contributory to a lack of OMS awareness, only 37 vocationally registered OMS surgeons are locally practising, presenting a surgeon-to-population ratio of 1:119,755 compared to Plastics (1:48,789), ENT (1:29,602), Ophthalmology (1:21,864) and Dental (1:1,686) [5, 6]. One-in-eight (12.8%) OMS surgeons work exclusively in public [7].

Previous studies have reported a limited understanding of the procedures performed by OMS and perception of the OMS SOP among medical and dental students, dentists, doctors and specialists [3, 8–13]. These studies have focused on assessing the perception of OMS relative to specific OMS scenarios and conditions via respondent referral and/or treatment preference, referral appropriateness and perceived competency or ability to treat a particular condition. There is a lack of literature that investigates the understanding of OMS by reporting on which speciality respondents believe manages different locoregionally relevant clinical scenarios or procedures. Furthermore, there is yet no study in NZ or internationally which investigates respondents understanding of the OMS SOP and the ability to differentiate OMS from similar specialties by analysing what respondents believe OMS does, does not do and how it is surgically distinguished from other specialties. Therefore, the study aims to explore the ability of Dental Students (DS), Medical Students (MS), Trainee Interns (TI) and Pre-vocational Junior Doctors (JD) in NZ in identifying procedures performed by OMS, the scope of OMS and their ability to differentiate OMS from similar specialities, to serve as a quantitative basis for dental and medical workforce training and improvement in patient care and healthcare efficiency. Although the practical NZ OMS SOP may differ from that internationally, the implications of a lack of understanding of OMS procedures and the inability to differentiate OMS from other specialities can impact overall OMS healthcare provision on a global level. The crucial growing pool of evidence that this study contributes to will provide support both regionally and internationally to reduce these discrepancies and provide equitable care in the OMS field overall.

The study hypothesis is that the understanding of the scope of OMS is limited however is greater among DS compared to MS, TI and JD. The second hypothesis is the ability among DS, MS, TI and JD to differentiate OMS from similar specialities is low.

## Materials and methods

### Ethical approval

The study was performed in line with the principles of the Declaration of Helsinki. Ethical approval was granted by the University of Otago Human Ethics Committee (Reference number D23/030).

### Study design, recruitment, inclusion and exclusion

A cross-sectional survey was performed. The study commenced April 2023 and concluded August 2023. Survey invitations were distributed electronically to participants via their respective University dental and medical schools and promoted in a junior doctor organisation e-newsletter. Participant information sheets were provided, and informed consent obtained. Participants included Dental students in 4th or 5th years, Medical Students in 4th or 5th years and Trainee Interns enrolled in a NZ dental or medical program and pre-vocational post-graduate years 1–3 (PGY1-3) Junior Doctors who are NZ medical school graduates currently practising in NZ. Medical students and doctors with dental degree/s and dental students with medical degree/s were excluded. An optional grocery voucher draw was offered for participation incentive.

### Questionnaire

An electronic questionnaire was developed by the research group in conjunction with a medical advisor and reviewed separately by two independent academic clinicians. The final survey consisted of 90 questions across three sections. The first section comprised 9 General Demographic questions. The next section contained 11 questions relating to Awareness of OMS, OMS career ambition, Teaching and Exposure to OMS, Confidence in identifying the scope of practice and procedures performed by OMS, ENT, Plastics, Ophthalmology and Dental and Confidence in referring to these specialities. A 5-point Likert scale was used for questions relating to OMS teaching and exposure and confidence in identifying and referring the OMS and similar specialities. The final section included 70 procedures across **four key domains**: (Domain 1) Cosmetics and Reconstruction, (Domain 2) Trauma, Urgent and Emergency Care, (Domain 3) Cancer and Pathology, and (Domain 4) General. Participants were asked to identify which specialty or specialities among OMS, ENT, Plastics, Ophthalmology and Dental performed each procedure.

Four key metrics were used including:

1. *Awareness of OMS training*: categorised as None, Low, Fair or Good based on number of correct responses to (a) identification of OMS as a dual medical and dental speciality, (b) identification of organisation responsible for OMS training, and (c) number of years required to qualify as an OMS surgeon from university commencement.
2. *Identification of procedures performed by OMS*: refers to correct identification of procedures performed by OMS.
3. *Identification of the OMS SOP*: refers to respondents who correctly identifies ≥ 70% of procedures performed by OMS and identifies ≤ 30% of non-OMS procedures as being performed by OMS.
4. *Differentiation of OMS from similar specialities* refers to respondents who correctly identifies the OMS SOP and correctly identifies ≥ 70% of procedures performed by ≥ 2 different specialities.

The same parameters were applied to respective specialities regarding identification and differentiation.

### Data collection and statistic analysis

Data was electronically collected via Qualtrics and all responses were exported in excel documents. Exported data was analysed with IBM SPSS version 29. A p-value of < 0.05 was considered statistically significant. Categorical variables were analysed using a Chi-square test.

## Results

Of 387 responses received, 282 (72.9%) were included. Responses were excluded if they did not disclose their level of medical training (*n* = 66) or were incomplete (*n* = 39). Table 1 shows participant characteristics and Supplementary Tables 1 and Table 2 provide participant responses to individual procedures and domains respectively.

### Participant characteristics

**Table 1:** Participant Characteristics

	MS	TI	JD	Medical	DS	Total
Participants *n (%)*						
	100 (35.5)	58 (20.6)	50 (17.8)	208 (73.7)	74 (26.2)	282 (100.0)
Age *years*						
Mean	23.6	24.4	26	24.8	24.2	24.7
Range	20 to 39	21 to 34	24 to 47	20 to 47	20 to 37	20 to 47
Gender *%*						
Female	62.0	70.6	46.9	60.8	62.1	61.2
Male	38.0	29.3	46.9	37.6	36.4	37.3
Non-binary	0.0	0.0	2.0	0.4	1.3	0.7
Prefer not to say/Unknown	0.0	0.0	4.0	0.9	0.0	1.0
Entry Pathway into University *%*						
Direct Entry	63.0	68.8	85.4	70.0	60.8	67.5
Post-graduate Entry	31.0	22.4	8.3	23.1	16.2	21.3
Other	6.0	8.6	6.2	6.7	22.9	11.0
Primary Caregiver Income Prior to Entering University Studies *%*						
Up to $14,000	12.1	1.7	0.0	6.2	10.8	7.4
From $14,000 to $48,000	6.0	10.3	6.1	6.7	4.0	6.0
From $48,000 to $70,000	22.2	32.7	2.0	17.8	6.7	14.9
From $70,000 to $180,000	28.2	65.5	44.8	33.3	29.3	32.3
Over $180,000	10.1	13.7	10.2	11.1	21.6	13.8
Prefer not to say/Unknown	21.2	20.6	36.7	24.6	27.0	25.2
Current Region of Training/Work with Advanced Trauma Unit *%*						
Advanced Trauma Unit	88.7	82.4	62.5	80.7	100.0	85.9
No Advanced Trauma Unit	11.2	17.5	37.5	19.2	0.0	14.0

**Table 2:** Awareness of OMS Training and Career Ambition

	MS	TI	JD	Medical	DS	Total
From commencement of university, how many years do you believe it takes to qualify as an OMS surgeon? *years*						
Mean	13.2	14.7	11.8	12.4	10.1	12.5
Median	14	15	12	13	10	13
Range	5 to 22	8 to 20	4 to 20	4 to 22	3 to 19	3 to 22
Do you consider OMS a specialty of medicine, dentistry or both? ***%***						
Medicine	17.2	5.2	10.2	12.1	0.0	8.9
Dentistry	5.1	5.2	4.1	4.9	10.8	6.4
Both	77.8	89.7	83.7	82.5	87.8	83.9
Don’t know	0.0	0.0	2.0	0.5	1.4	0.7
What institute is responsible for training OMS surgeons in Australia and New Zealand? *%*						
University of Auckland	0.0	0.0	6.1	1.4	0.0	1.2
University of Otago	6.0	0.0	8.1	4.8	14.9	8.1
**ANZAOMS	9.0	3.4	6.1	6.7	10.8	8.7
**ANZCOMS (fictional organisation)	43.0	41.3	36.7	41.0	18.9	33.7
**RACS	5.0	5.1	8.1	5.7	4.0	6.5
**RACDS	7.0	10.3	6.1	7.7	17.5	9.6
You have to travel outside of Australia/New Zealand	0.0	1.7	0.0	0.4	0.0	0.6
Don’t know	30.0	37.9	28.5	31.8	33.7	31.2
Awareness of OMS specialty training *%*						
None	19.0	6.8	14.0	21.8	6.7	18.6
Low	52.0	58.6	58.0	50.3	74.3	55.4
Fair	27.0	27.5	28.0	25.5	14.8	23.2
Good	2.0	6.8	0.0	2.1	4.0	2.5
Mean score (with p-value)	1.1	1.3	1.1	1.0	1.1	1.0
How likely are you to pursue a careeer in OMS? *%*						
None	24.2	39.6	46.9	34.2	18.9	30.7
Low	57.5	50.0	40.8	51.0	36.4	47.6
Fair	12.1	10.3	8.1	10.2	29.7	14.7
Moderate	3.0	0.0	2.0	2.8	12.1	5.0
High	3.0	0.0	2.0	1.6	2.7	1.8
Mean score (with p-value)	2.0	1.6	1.7	1.8	2.4	1.9
MS	.	0.083	0.165	.	0.014*	.
TI	.	.	0.998	.	< 0.001*	.
JD	.	.	.	.	< 0.001*	.
Medical	.	.	.	.	< 0.001*	.
Do you believe you woul be more likely to pursue a career in OMS if you had more teaching and exposure during your dental (medical) training or PGY1-3 years? *%*						
Yes	55.5	55.1	42.8	52.6	67.5	56.1
No	20.2	20.6	18.3	20.4	18.9	20.0
Unsure	24.2	24.1	38.7	26.9	13.5	23.8

*Statistically significant. **ANZAOMS (Australian and New Zealand Association of Oral and Maxillofacial Surgery), ANZCOMS (Australian and New Zealand College of Oral and Maxillofacial Surgery - a fictitious organisation), RACS (Royal Australasian College of Surgeons), RACDS (Royal Australasian College of Dental Surgeons)

The study included 74 (26.2%) DS and 208 (73.7%) medical respondents, of which there were 100 (35.5%) MS, 58 (20.6%) TI and 50 (17.8%) JD. Mean respondent age was 24.7 years, ranging from 20 to 47 years. Three-fifths (61.2%) were female. Respondents identified as NZ European (49.6%), Chinese (21.2%), Māori (10.3%), Indian (3.9%), Pasifika (3.9%) and Other (21.3%). Two-thirds (67.5%) entered medicine or dentistry via direct-entry, one-fifth (21.3%) were post-graduates and 11.0% were alternative-pathway entrants. All DS and 58.0% of medical respondents attended or were attending the University of Otago. Most (85.9%) respondents were located within regions with an Advanced Trauma Centre, of which were mainly Canterbury (39.0%) or Auckland (25.6%). No significant difference was observed between groups regarding age, gender, ethnicity, entrance pathway or region of study/work.

### Awareness of OMS specialty training

Overall OMS awareness was low (1.0), with no significant difference between the groups (Table 2). Total perceived required OMS training time from commencing university ranged from 3 to 22 years, with a mean of 12.5 years (Table 2). 23.3% believed training time was 15–16 years, whilst 45.7% believed this was ≤ 12 years. Most (83.9%) respondents identified OMS as a dual specialty of medicine and dentistry (Table 2). Few (9.6%) respondents identified the Royal Australasian College of Dental Surgeons (RACDS) as the organisation responsible for OMS training, whilst most (33.7%) believed training was provided through the fictitious Australian and New Zealand College of Oral and Maxillofacial Surgery (Table 2). Participants with Good awareness identified a greater proportion of procedures performed by OMS (64.1% vs. 24.3%, *p* < 0.001) (Table 3), ENT (59.4% vs. 27.4%, *p* = 0.002), Plastics (63.6% vs. 30.6%, *p* = 0.002), Ophthalmology (76.5% vs. 38.5%, *p* = 0.003) and Dental (70.2% vs. 33.4%, *p* = 0.002) compared to those with None awareness. Good awareness was associated with a better ability to identify the OMS SOP (*p* < 0.001) and differentiate OMS from similar specialities (*p* < 0.001), compared to other groups (Table 3).

**Table 3:** Teaching and Exposure to OMS, Confidence in Identifying and Referring to OMS, Awareness of OMS Training vs. Identification of Procedures performed by OMS, OMS SOP Identification and Differentiation of OMS from Similar Specialties

	Identification of OMS Procedures					Identification of OMS SOP					Differentiation of OMS from similar specialties				
Teaching and Exposure to OMS During Dental/Medical School (mean %, with p-value)															
	None	Small	Fair	Moderate	Significant	None	Small	Fair	Moderate	Significant	None	Small	Fair	Moderate	Significant
Mean %	44.2	47.0	59.6	73.4	0.0	6.3	6.5	22.7	0.0	0.0	3.2	5.4	9.1	0.0	0.0
None	.	0.748	0.028*	0.152	.	.	1.000	0.036*	.	.	.	0.819	0.624	0.994	.
Small	.	.	0.930	0.224	.	.	.	0.034*	.	.	.	.	0.866	0.973	.
Fair	.	.	.	0.777	.	.	.	.	.	.	.	.	.	0.899	.
Moderate	.	.	.	.	.	.	.	.	.	.	.	.	.	.	.
Significant	.	.	.	.	.	.	.	.	.	.	.	.	.	.	.
Confidence in identifying the scope of practice and types of procedures performed by OMS (mean %, with p-value)															
	None	Low	Fair	Moderate	High	None	Low	Fair	Moderate	High	None	Low	Fair	Moderate	High
Mean %	37.2	43.2	55.2	62.7	60.2	2.9	4.4	8.0	30.0	37.5	0.0	3.3	2.7	20.0	37.5
None	.	0.627	0.002*	< 0.001*	0.840	.	0.998	0.871	0.002*	0.005*	.	0.907	0.969	0.005*	< 0.001*
Low	.	.	0.002*	0.003*	0.249	.	.	0.839	< 0.001*	0.003*	.	.	0.999	0.005*	< 0.001*
Fair	.	.	.	0.693	0.977	.	.	.	.	.	.	.	.	0.007*	< 0.001*
Moderate	.	.	.	.	0.999	.	.	.	.	.	.	.	.	.	0.236
High	.	.	.	.	.	.	.	.	.	.	.	.	.	.	.
Confidence in referring to OMS (mean %, with p-value)															
	None	Low	Fair	Moderate	High	None	Low	Fair	Moderate	High	None	Low	Fair	Moderate	High
Mean %	41.6	43.4	53.0	59.8	55.4	4.4	1.9	13.3	16.1	28.6	0.0	1.9	6.7	9.7	28.6
None	.	0.989	0.186	0.023*	0.219	.	0.979	0.351	0.290	0.019*	.	0.980	0.419	0.257	< 0.001*
Low	.	.	0.005*	< 0.001*	0.072	.	.	0.015*	0.041*	0.002*	.	.	0.476	0.310	< 0.001*
Fair	.	.	.	0.650	0.931	.	.	.	0.986	0.247	.	.	.	0.959	0.003*
Moderate	.	.	.	.	1.000	.	.	.	.	0.557	.	.	.	.	0.036*
High	.	.	.	.	.	.	.	.	.	.	.	.	.	.	.
Awareness of OMS Specialty Training (mean %, with p-value)															
	None	Low	Fair	Good		None	Low	Fair	Good		None	Low	Fair	Good	
Mean %	24.3	46.8	47.7	64.1		1.5	7.8	4.9	44.4		0.0	4.7	2.5	44.4	
None	.	< 0.001*	< 0.001*	< 0.001*		.	0.292	0.840	< 0.001*		.	0.331	0.825	< 0.001*	
Low	.	.	0.994	0.170		.	.	0.821	< 0.001*		.	.	0.825	< 0.001*	
Fair	.	.	.	0.231		.	.	.	< 0.001*		.	.	.	< 0.001*	
Good	.	.	.	.		.	.	.	.		.	.	.	.	

*Statistically significant.

### OMS teaching and exposure to during medical or dental training and PGY1-3 years

Most (92.2%) respondents reported having None to Small amount of OMS teaching and exposure during medical or dental school (Table 4). One-quarter (24.3%) of dental respondents reported having Fair-Moderate among of OMS teaching and exposure compared to medical respondents (2.9%) (Table 4). All respondents except 4 preferred to have more OMS teaching and exposure than they had experienced, with 61.8% preferring a Fair to Moderate amount. Participants with greater OMS teaching and exposure were more confident in identifying and referring to OMS (Table 4), identified a greater proportion of procedures performed by OMS, were able to better identify the OMS SOP and differentiate OMS from similar specialities (Table 4).

### OMS career ambition

Most (78.3%) reported No to Low likelihood to pursue a career in OMS (Table 2). DS were significantly more likely to pursue a career in OMS compared to medical respondents (2.4 vs. 1.8, *p* < 0.001) (Table 2). Participants with greater OMS exposure and teaching during dental/medical school were more likely to pursue a career in OMS, however this was not statistically significant. 56.1% believed they would be more likely to pursue a career in OMS if they had more OMS teaching and exposure during their dental/medical degree or PGY1-3 years (Table 2).

Those with a High (75.0%) or Fair (57.3%) likelihood to pursue OMS as a career were better at identifying procedures performed by OMS compared to Low (44.5%, *p* = 0.012 and *p* = 0.005) or No (43.7%, *p* = 0.018 and *p* = 0.019) raters. High likelihood to pursue a career in OMS was associated with both a significantly better ability to identify the scope of OMS and differentiate it from similar specialities, compared to a Moderate (*p* = 0.022, *p* = 0.001), Fair (*p* = 0.002, *p* < 0.001), Low (*p* < 0.001, *p* < 0.001) or No (*p* = 0.001, *p* < 0.001) likelihood.

### Confidence in identifying the scope of OMS and similar specialities

Respondents were less confident in identifying OMS compared to similar specialities (Table 4). 67.7% reported No to Low confidence in identifying OMS compared to 32.4% for similar specialities (Table 4). Significantly greater confidence in identifying the OMS SOP was observed among JD and DS compared to TI (*p* < 0.001, *p* = 0.002) and MS (*p* < 0.001, *p* = 0.007) (Table 4). Greater confidence in identifying OMS SOP was associated with identification of a greater proportion of procedures performed by OMS, greater ability to identify the OMS SOP and differentiate OMS from similar specialities (Table 3).

JD were significantly more confident in identifying other specialities compared to other groups (Table 4), whereas DS were significantly less confident than TI (*p* = 0.004) and MS (*p* = 0.007). No statistical difference was observed among confidence in identifying the scope of ENT, Plastics, Ophthalmology and Dental and ability to differentiate these specialities.

### Confidence in referring to OMS and similar specialities

Two-thirds (62.3%) or respondents reported No to Low confidence in referring to OMS compared to one-third (33.1%) for similar specialities (Table 4). JD and DS were significantly more confident than TI and MS in referring to OMS (*p* < 0.001) (Table 4). All groups showed equivalent or greater confidence in identifying the OMS SOP than referring to OMS, except JD (Table 4). Greater confidence in referring to OMS was associated with greater identification of procedures performed by OMS, the OMS SOP and differentiating OMS from similar specialities (Table 3). JD were significantly more confident in referring to similar specialities compared to MS (*p* < 0.001), TI (*p* = 0.002) and DS (*p* < 0.001) (Table 4). No statistical difference was observed among confidence in referring to ENT, Plastics, Ophthalmology and Dental and ability to differentiate these specialities. Medical groups were more confident in referring to similar specialities than to OMS (Table 4).

### Stage of professional training

DS recognised more procedures performed by OMS (66.5%), Ophthalmology (75.3%) and Dental (81.8%) compared to other groups and was significant across all domains for OMS (*p* < 0.001) and Dental (*p* < 0.001) (Table 2). TI recognised more procedures performed by ENT (62.2%) and Plastics (62.3%) than other groups (Table 2). Medical respondents identified significantly more ENT procedures compared to DS (*p* < 0.001) (Table 2). DS were significantly better at identifying the OMS SOP compared to medical respondents (14.9 vs. 6.3, *p* = 0.002) however this was not significant between subgroups (Supplementary Table 3). DS were significantly better at identifying the Dental SOP compared to other groups (*p* < 0.001) however worse than MS and TI at identifying the ENT SOP (*p* = 0.047 and *p* < 0.001) (Supplementary Table 3). No significant difference was observed among medical groups regarding identification of procedures performed by OMS or the OMS SOP.

### Differentiating OMS and similar specialities

Differentiation of OMS from similar specialities was lowest (5.0), followed by ENT (6.8), Plastics (11.1), Dental (15.4) and Ophthalmology (20.9) (Supplementary Table 2). DS were significantly better at differentiating OMS from similar specialities compared to medical respondents (12.2 vs. 2.2, *p* < 0.001) (Supplementary Table 2). No statistical difference regarding differentiation of OMS from similar specialities was observed between medical groups. No statistically significant difference was observed among groups regarding differentiation of ENT or Plastics. DS (37.8) were significantly better at differentiating Ophthalmology from similar specialities compared to MS (12.0, *p* < 0.001), TI (13.8, *p* = 0.003) and medical respondents (10.9, *p* < 0.001) (Supplementary Table 2). DS (33.8) were significantly better at differentiating Dental from similar specialities compared to MS (9.0, *p* < 0.001), TI (6.9, < 0.001) and JD (12.0, *p* = 0.004). No statistical difference was observed among medical groups in differentiating any specialty.

### OMS procedures

The most recognised clinical situations managed by OMS were mandible fracture (95.4%), complex dentoalveolar fractures (93.3%), temporomandibular joint replacement (93.3%), debridement of osteomyelitis of the mandible (91.1%), cosmetic mandibular recontouring (89.7%), odontogenic keratocyst surgery (89.7%), temporomandibular joint arthrocentesis (89.7%) and orthognathic surgery (88.3%) (Supplementary Table 2). Dental implants and bone grafting (65.6%) and third molar surgery (55.3%) were poorly recognised.

Urgent and Emergency procedures performed by OMS were variably recognised, including an acute bleed from a LeFort II mid-face fracture (86.5%), facial space infections (77.0%), orbital floor fracture (70.2%), tracheostomy/surgical access for an emergency airway (47.2%) and surgical management of a traumatic retrobulbar haemorrhage (33.7%) (Supplementary Table 2).

Dental procedures incorrectly identified as performed by OMS were frequent, including broken denture repair (24.1%), anterior dental veneers (23.0%), pre-bisphosphonate dental checks (20.9%), orthodontics (17.7%), periodontal debridement (12.4%), removal of dental decay and placing a filling (11.3%) and teeth whitening (5.7%). (Supplementary Table 2).

Procedures performed by OMS were recognised only half the time (52.9%) (Supplementary Table 3). Trauma, Urgent and Emergency Care procedures were most recognised (61.5%) whilst Cosmetic and Reconstruction was least recognised (35.5%) (Supplementary Table 3).

## Discussion

The present study highlighted an overall low ability among Dental Students (DS), Medical Students (MS), Trainee Interns (TI) and Pre-vocational Junior Doctors (JD) to identify procedures performed by OMS, the OMS SOP and ability to differentiate OMS from similar specialities, however DS were more able compared to medical respondents. OMS was least recognised among all specialities.

### Ability to differentiate OMS from similar specialities

To the authors knowledge, this is the first study to report on the ability to differentiate OMS from similar specialities. The ability to differentiate requires recognising what a specialty does, does not do and distinguish it from another. Differentiation of OMS was lowest among the different specialities. The greater ability in differentiating OMS from similar specialities among DS may be explained by the continual clinical and academic exposure to OMS, staff, procedures, and teaching during dental training. Unexpectedly, progressive improvement among MS, TI and JD in identifying procedures and the SOP of ENT, Plastics, Ophthalmology and Dental was not observed. Explanations include a potential down-stream effects of COVID-19 on medical training, a pre-vocational focus on the general scope of medicine rather than subspecialist areas and a lack of OMS continuing medical education. Unexpectedly, DS were significantly more likely to differentiate Ophthalmology from similar specialities compared to medical respondents, which may reflect the current medical education framework within the dental curriculum, including a multi-disciplined approach incorporating locoregional head and neck education, albeit Ophthalmologists are not involved in dental training. Furthermore, medical curricula focus on training competent generalist junior doctors rather than specialised doctors and therefore exposure to specialities may be limited.

### Scope of practice

Studies vary in how they investigate the perception of OMS SOP, predominantly focusing on *treatment or referral preference* [3, 11, 13–25], *referral appropriateness* [26–30] and *perceived specialty competency specialty/ability to treat* [12, 15, 20, 26, 27, 31, 32]. This study applied a similar procedural-based SOP approach however contrastingly, it investigated participant perception by asking which specialty/specialities performed each clinical procedure/scenario to minimise potential confounding factors. For example, referral preference may reflect local professional relationships rather than the perceived SOP.

### Why is OMS so poorly understood?

In 1977, the specialty name changed from *Oral Surgery* to *Oral and Maxillofacial Surgery* with the intent to clarify the scope of the profession [33]. The changed name has also been labelled as lengthy and confusing due to Latin etymology [11]. Although potentially applicable to the general public, Latin and Greek terminology are embedded in healthcare professional training and has unlikely confused referrals to Greek-derived named specialities such as Orthopaedics or Cardiology. The ongoing interchangeable use of *Oral Surgeon* and *Oral and Maxillofacial Surgeon* has continued the confusion, particularly within the dental field [34]. Furthermore, the intra-professional division among acronym usage varies from the prevalent and preferred OMS to OMFS, potentially contributing to confusion [35]. There has been a historic fluctuation in connectivity between the medical profession and oral cavity for various reasons [36]. The absence of formal OMS teaching within the contemporary medical curricula is reflected by the findings of this study.

There is a perception among OMS that general practitioners and hospital doctors have a poor understanding of the OMS SOP [2]. There is limited understanding of the OMS SOP across all levels globally [3, 10–14, 16–18, 20, 24, 26, 32]. The current study supports this, with only half of procedures performed by OMS being recognised as such. Contrastingly, a Brazilian study of doctors, dentists, medical and dental students found a good level of understanding of the OMS SOP [19] whilst an Australian study of General Practitioners identified adequate awareness of the core OMS fields [22]. A NZ study investigating maxillofacial trauma identified a good knowledge for OMS referral among General Practitioners regarding maxillofacial trauma [25].

This study concurred with others [15, 29] in that undergraduate teaching and exposure to OMS was limited. The University of Otago dental curricula includes multi-year teaching with an opportunity for clinical exposure whilst only some NZ medical schools include a possible short-term exposure. This may explain the greater awareness of OMS observed among the dental field [13, 20, 28]. Similarly, this study demonstrated a greater ability of DS compared to medical respondents in recognising procedures performed by OMS, the SOP and ability to differentiate OMS from similar specialities. Despite little interest in pursuing an OMS career [37], the study found most participants desired more teaching and exposure to OMS during undergraduate years than they had experienced and identified a clear need. A NZ study reported General Practitioners recognising the importance of continued education regarding maxillofacial injuries [25]. The OMS public presence in NZ hospitals is comparatively less than other specialities, with a recent study finding only 5 OMS surgeons worked exclusively in public [7]. Despite a relatively low numerical presence, four of the five NZ Advanced Trauma Centres have attached OMS training units. These Advanced Trauma Centres also have strong medical school educational relationships, representing an opportunity. Furthermore, with the recent exception of one hospital, only dental-qualified applicants are employed in maxillofacial house surgeon posts. The result highlights a referral workforce with limited confidence, exposure and understanding of OMS.

### Awareness of OMS training

A recent Australian study reported a limited understanding of OMS training among DS [38]. The current study identified similar findings across DS, MS, TI and JD. Those with a high awareness also reported a high likelihood to pursue a career in OMS and are therefore unlikely to be future referrers. Interestingly, respondents mainly identified the fictitious Australian and New Zealand College of Oral and Maxillofacial Surgery as the organisation responsible for OMS training, rather than the Royal Australasian College of Dental Surgeons. Although incorrect, this may reflect an awareness that OMS is separate from Dentistry and that formal training is undertaken by a College, similar to other surgical specialities.

### Further areas

In NZ, OMS provides the primary specialist level of care for complex maxillofacial trauma whilst General Practitioners and Emergency Care doctors provide basic care. The current study highlights a greater awareness of the role of OMS regarding maxillofacial trauma compared to other maxillofacial areas within the SOP. Contrary to perceptions within the OMS field [2], a NZ study investigating maxillofacial trauma identified a good knowledge for OMS referral among general dentists regarding maxillofacial trauma [25].

The current study highlights a poor understanding among dental and medical respondents of the surgical skillset OMS offers in cosmetic facial surgery, and is similarly reported elsewhere [22, 23, 26, 27]. This is likely multifactorial, including commercial and volumetric procedural dominance from other specialities, social media, a lack of education and understanding of OMS and specialty presence in other areas including TMJ, orthognathics and oncology surgery. Other poorly recognised areas include obstructive sleep apnoea surgery, cleft lip and palate surgery and head and neck oncology.

The OMS role in time-critical emergency events appears poorly understood and may harm patient outcomes. Concerningly, this is least understood among medical respondents who are most likely to acutely assess and refer these patients. Orbital fractures and traumatic retrobulbar haemorrhage may threaten life and vision in the context of oculocardiac reflex and orbital compartment syndrome. Facial space infections may catastrophically compromise the airway or lead to haemodynamic instability through sepsis. Establishment of a surgical emergency airway is also paramount to life continuity. Albeit Emergency Care doctors usually provide initial supportive management including resuscitation, non-definitive airways, and vasopressor support.

### Specialty confusion and a referral pattern change

The study highlighted the ongoing confusion between Dental and OMS, particularly among medical respondents. OMS was frequently identified as performing dental procedures. The historic basis of OMS, overlap among OMS and Dental SOP, absence of OMS teaching in medical curricula and limited clinical exposure may compound this. Furthermore, the RACDS awards the registrable FRACDS(OMS) qualification rather than the Royal Australasian College of Surgeons or a standalone specialty college as with other surgical specialities. Dental implants, bone grafts and third molar surgery are the mainstay of private OMS practice, yet only half of respondents believed OMS performed these procedures. Unexpectedly more dental respondents identified these procedures as performed by Dental compared to OMS, which contrasts the literature [13, 16, 17, 21, 28, 31]. As most referrals for dental implants and third molar surgery originate from dentists, this may identify an emerging referral pattern change. This is likely multifactorial reflecting an increasing confidence in performing oral surgical procedures among dentists, increased implant and surgical training at undergraduate level, increased economic and patient demand, increased exposure and education from dental specialist Oral Surgeons and low OMS surgeon numbers. The OMS adage “*dentoalveolar surgery can always humble you*” should be highlighted, with a potential for increased acute public system referrals in the context of post-operative issues.

The greatest finding of this study is that it demonstrated (1) a poor ability in identifying and differentiating OMS, (2) a clear desire to have more education, and (3) a clear benefit in providing previous exposure and training during undergraduate years.

**Table 4:** OMS teaching and exposure, Confidence in identifying procedures, SOP and Referring to OMS and Similar Specialties

	MS	TI	JD	All Medical	DS	Overall	MS	TI	JD	All Medical	DS	Overall
During medical/dental school, how much Teaching and Exposure to OMS:												
	Q3. Did you have (have you had)? *%*						Q4. Would you prefer (had preferred)? *%*					
None	60.6	39.6	51.0	49.3	6.7	39.4	3.0	0.0	0.0	1.6	0.0	1.2
Small amount	37.3	56.8	44.8	47.7	68.9	52.6	44.4	43.1	38.7	40.0	5.4	31.9
Fair amount	2.0	1.7	2.0	2.0	22.9	6.8	41.4	44.8	53.0	47.7	40.5	46.0
Moderate amount	0.0	1.7	2.0	0.8	1.3	0.9	10.1	12.0	4.0	8.5	39.1	15.6
Significant amount	0.0	0.0	0.0	0.0	0.0	0.0	1.0	0.0	4.0	2.0	14.8	5.0
Mean score (with p-value)	1.4	1.6	1.5	1.5	2.1	1.6	2.6	2.6	2.7	2.6	3.6	2.9
MS	.	0.060	0.530	.	< 0.001*	.	.	0.934	0.801	.	< 0.001*	.
TI	.	.	0.791	.	< 0.001*	.	.	.	0.990	.	< 0.001*	.
JD	.	.	.	.	< 0.001*	.	.	.	.	.	< 0.001*	.
Medical	.	.	.	.	< 0.001*	.	.	.	.	.	< 0.001*	.
Rate your confidence in identifying the scope of practice and types of procedures performed by:												
	Q5. OMS *%*						Q6. ENT, Plastics, Ophthalmology and Dental *%*					
None	16.1	18.9	2.0	13.4	1.3	10.6	4.0	3.5	0.0	3.2	5.4	3.7
Low	60.6	60.3	44.8	59.1	50.0	57.1	26.2	22.8	14.2	22.9	47.9	28.7
Fair	15.1	17.2	34.6	19.1	37.8	23.5	36.3	35.0	30.6	33.6	30.1	32.8
Moderate	5.0	1.7	12.2	5.3	9.4	6.2	27.2	33.3	28.5	30.7	16.4	27.4
High	3.0	1.7	6.1	2.8	1.3	2.5	6.0	5.2	26.5	9.4	0.0	7.2
Mean score (with p-value)	2.1	2.0	2.7	2.2	2.5	2.3	3.0	3.1	3.6	3.2	2.5	3.0
MS	.	0.706	< 0.001*	.	0.008*	.	.	0.940	0.001*	.	0.007*	.
TI	.	.	< 0.001*	.	< 0.001*	.	.	.	0.021*	.	0.004*	.
JD	.	.	.	.	0.694	.	.	.	.	.	< 0.001*	.
Medical	.	.	.	.	0.002*	.	.	.	.	.	< 0.001*	.
Rate your confidence in referring to:												
	Q7. OMS *%*						Q8. ENT, Plastics, Ophthalmology and Dental *%*					
None	23.2	20.6	2.0	16.7	5.4	14.1	8.0	6.8	0.0	6.9	16.2	9.0
Low	60.6	55.1	22.4	52.2	35.1	48.2	23.2	13.7	6.1	17.9	44.6	24.1
Fair	8.1	18.9	44.8	19.1	37.8	23.5	44.4	43.1	28.5	39.5	28.3	36.9
Moderate	4.0	5.1	20.4	7.7	16.2	9.7	19.1	31.0	44.8	26.9	9.4	22.8
High	4.0	0.0	10.2	4.0	5.4	4.3	5.0	5.1	20.4	8.5	1.3	6.8
Mean score (with p-value)	2.0	2.0	3.1	2.2	2.8	2.4	2.8	3.1	3.7	3.1	2.3	2.9
MS	.	1.000	< 0.001*	.	< 0.001*	.	.	0.410	0.001*	.	< 0.001*	.
TI	.	.	< 0.001*	.	< 0.001*	.	.	.	0.002*	.	< 0.001*	.
JD	.	.	.	.	0.190	.	.	.	.	.	< 0.001*	.
Medical	.	.	.	.	< 0.001*	.	.	.	.	.	< 0.001*	.
Teaching and Exposure to OMS during medical/dental school compared with confidence in:												
	Identifying the SOP and procedures performed by OMS *%*						Referring to OMS *%*					
Amount	None	Small	Fair	Moderate	Significant	Overall	None	Small	Fair	Moderate	Significant	Overall
None	19.0	6.0	0.0	0.0	0.0	10.7	19.0	11.3	9.1	0.0	0.0	14.1
Low	57.9	60.1	31.8	33.3	0.0	57.1	54.8	47.6	22.7	0.0	0.0	48.3
Fair	16.7	23.8	59.1	33.3	0.0	23.5	15.1	28.0	36.4	33.3	0.0	23.5
Moderate	3.2	7.7	9.1	33.3	0.0	6.3	7.9	8.3	27.3	33.3	0.0	9.7
High	3.2	2.4	0.0	0.0	0.0	2.5	3.2	4.8	4.5	33.3	0.0	4.4
Mean score (with p-value)	2.1	2.4	2.8	3.0	0.0	2.3	2.2	2.4	2.9	4.0	0.0	2.4
None	.	0.030*	0.005*	0.279	.	.	.	0.100	0.006*	0.009*	.	.
Small amount	.	.	0.204	0.604	.	.	.	.	0.130	0.036*	.	.
Fair amount	.	.	.	0.970	.	.	.	.	.	0.296	.	.
Moderate amount	.	.	.	.	.	.	.	.	.	.	.	.
Significant amount	.	.	.	.	.	.	.	.	.	.	.	.

*Statistically significant

### How do we address this?

A multimodal approach can be taken to improve the understanding of OMS among dental and medical colleagues. Integration of OMS teaching within the medical curricula, including emergency situations provides an academic introduction to the speciality as seen with other medical disciplines. OMS placements for dental and medical students would provide a relevant clinical context to reinforce their academic understanding. A 2–4 week OMS placement would be appropriately comparable with other specialties, whilst respecting a pragmatic balance with the requirements to train competent general junior dentists and doctors. Encouraging active involvement of the OMS profession with continued professional education for junior and senior doctors and dentists may opportunistically maximise both professional awareness and OMS understanding. This may take place at conferences, trauma courses, and presentations. Encouraging recruitment of both medical-qualified and dental-qualified maxillofacial house officers would provide a complimentary skillset for patient care, maximise interprofessional relationships and increase awareness and understanding among both groups. Reorientating the OMS profession to have an increased presence in the public sector would increase public and professional exposure and bolster service provision and may include postings at advanced trauma centres, university-associated units and involvement in organisations.

### Study weaknesses and future proposals

Participants who consented to participate may have had a higher level of understanding of OMS SOP than those who did not, therefore the estimate of this impacted may be higher than anticipated. A larger sample size would further increase the power and ability for further subgroup analysis. A further weakness includes 66 excluded medical responses due to participants choosing not to disclose their level of training. Analysis with these excluded responses emphasised a greater difference between medical and dental respondents in all four key metrics, suggesting the overall understanding of procedures performed by OMS, the OMS SOP and ability to differentiate OMS from similar specialities is lower than what is reported. It is unclear whether inclusion would influence a progressive difference between among MS, TI and JD. 39 (10.1%) study responses were excluded due to incompleteness. The above study involved participants whose clinical and academic training may have been impacted by the COVID-19 pandemic which may have resulted in an overall lower recognition rate of specialities. The study was promoted in a junior doctor organisation e-newsletter which may have impacted respondent type and resulted in more favourable identification of the surgical specialities. A repeat future study would provide a response to the proposed Action Plan however may reflect a recovery in professional education from the COVID-19 impact. A further extension of the study to explore current referrers including Dentists, General Practitioners, Emergency Department doctors and other specialities would be useful.

### Key findings

- Limited ability to identify procedures performed by OMS.
- Limited ability to identify the OMS SOP.
- Limited ability to differentiate OMS from similar specialities.
- Low awareness of OMS role in potentially life- and/or vision- threatening Urgent and Emergency procedures/clinical scenarios, particularly among medical respondents.
- Low confidence in identifying OMS SOP.
- Low confidence in referring to OMS.
- Low perceived OMS teaching and exposure in undergraduate and early post-graduate years.
- Desire for greater OMS teaching and exposure in undergraduate and early post-graduate years.
- Confusion among OMS role in third molar surgery, implantology and bone grafts.
- Increased OMS teaching and exposure improves overall understanding of OMS.

## Conclusion

The current study has highlighted a deficit in the understanding of OMS among DS, MS, TI and JD with potential implications in the public and private healthcare sector. Identification of procedures, OMS SOP and ability to differentiate OMS from similar specialities is limited however slightly better among DS.

### Electronic supplementary material

Below is the link to the electronic supplementary material.

Supplementary Material 1

## Data Availability

No datasets were generated or analysed during the current study.

## Declarations

### Clinical trial number

This study was not a clinical trial, therefor no trial number available.
